# Expanding contraceptive options for PMTCT clients: a mixed methods implementation study in Cape Town, South Africa

**DOI:** 10.1186/1742-4755-11-3

**Published:** 2014-01-10

**Authors:** Theresa Hoke, Jane Harries, Sarah Crede, Mackenzie Green, Deborah Constant, Tricia Petruney, Jennifer Moodley

**Affiliations:** 1 FHI 360, 359 Blackwell Street, Durham, North Carolina 27701, USA; 2 Women’s Health Research Unit, School of Public Health & Family Medicine, University of Cape Town, Observatory 7925, Cape Town, South Africa

**Keywords:** PMTCT, Family planning, Intrauterine device, Female sterilization, Implementation, South Africa

## Abstract

**Background:**

Clients of prevention of mother-to-child transmission (PMTCT) services in South Africa who use contraception following childbirth rely primarily on short-acting methods like condoms, pills, and injectables, even when they desire no future pregnancies. Evidence is needed on strategies for expanding contraceptive options for postpartum PMTCT clients to include long-acting and permanent methods.

**Methods:**

We examined the process of expanding contraceptive options in five health centers in Cape Town providing services to HIV-positive women. Maternal/child health service providers received training and coaching to strengthen contraceptive counseling for postpartum women, including PMTCT clients. Training and supplies were introduced to strengthen intrauterine device (IUD) services, and referral mechanisms for female sterilization were reinforced. We conducted interviews with separate samples of postpartum PMTCT clients (265 pre-intervention and 266 post-intervention) to assess knowledge and behaviors regarding postpartum contraception. The process of implementing the intervention was evaluated through systematic documentation and interpretation using an intervention tracking tool. In-depth interviews with providers who participated in study-sponsored training were conducted to assess their attitudes toward and experiences with promoting voluntary contraceptive services to HIV-positive clients.

**Results:**

Following the intervention, 6% of interviewed PMTCT clients had the desired knowledge about the IUD and 23% had the desired knowledge about female sterilization. At both pre- and post-intervention, 7% of clients were sterilized and IUD use was negligible; by comparison, 75% of clients used injectables. Intervention tracking and in-depth interviews with providers revealed intervention shortcomings and health system constraints explaining the failure to produce intended effects.

**Conclusions:**

The intervention failed to improve PMTCT clients’ knowledge about the IUD and sterilization or to increase use of those methods. To address the family planning needs of postpartum PMTCT clients in a way that is consistent with their fertility desires, services must expand the range of contraceptive options to include long-acting and permanent methods. In turn, to ensure consistent access to high quality family planning services that are effectively linked to HIV services, attention must also be focused on resolving underlying health system constraints weakening health service delivery more generally.

## Background

Current global [1-4] and national [5] policies offer unprecedented support for linkages between family planning and HIV/AIDS services. The two largest global HIV/AIDS initiatives--The Global Fund to Fight AIDS, Tuberculosis and Malaria, and the President’s Emergency Plan for AIDS Relief (PEPFAR)—have increased financial support for programming to serve the reproductive health needs of women living with HIV/AIDS [3]. The evidence base for delivering integrated services is also growing. Studies have documented successful interventions in the context of HIV testing and counseling [6,7], antiretroviral treatment [8], and HIV prevention trials [9,10]. Yet there are few proven service delivery models to fill the family planning needs of postpartum PMTCT clients [11,12]. Research has shown that when postpartum HIV-positive women do use contraception, they rely chiefly on short-acting methods like condoms, pills, and injectables, even when reporting they wanted no future pregnancies [13-15].

Long-acting methods, like the intrauterine device (IUD) and contraceptive implants, and permanent methods, like female sterilization, offer superior contraceptive protection and convenience. Women are not required to visit health facilities regularly for re-supply, and they do not face adherence challenges that thwart the effectiveness of short-acting methods [16,17]. Yet few studies have examined strategies for expanding the range of contraceptive options for PMTCT clients and other HIV-positive women to include long-acting and permanent methods. One exception is a trial conducted by Stephenson and colleagues with HIV serodiscordant and concordant positive couples in Zambia [6]. Exposure to a video highlighting IUD and contraceptive implants was associated with greater choice of those methods. Another study conducted in Rwanda showed high uptake of implants when this method was made available to postpartum PMTCT clients [18].

Evidence is still needed on strategies for delivering long-acting and permanent contraceptive methods to postpartum PMTCT clients in the context of routine service delivery. South Africa is an appropriate context to conduct related research given the very high HIV prevalence; a 2010 national survey revealed 30% of pregnant women were HIV-positive [19]. Contraceptive use is also high; the most recent national estimate of contraceptive prevalence is over 60%, with injectable contraceptives dominating the method mix [20]. At the time the study was conducted, the government of South Africa had initiated policy discussions about linking HIV and family planning services [21], but clear guidance for service integration was largely absent.

This study, conducted in 2009–2010 in public sector facilities in Cape Town, South Africa, tested an intervention for promoting to PMTCT clients an expanded range of contraceptive options that included female sterilization and the IUD. (Contraceptive implants were not offered in public facilities at the time of the study.) Here we report on knowledge, attitudes, and behaviors related to contraceptive use among two samples of postpartum PMTCT clients surveyed before and after intervention implementation. We further report on the process of implementing the intervention, revealing the factors supporting and impeding delivery of health services aimed at serving the contraceptive needs of postpartum HIV-positive women.

## Methods

We conducted a mixed-methods study to test an intervention for expanding contraceptive options for postpartum PMTCT clients. The study’s primary outcome, examined for both the IUD and female sterilization, was the proportion of postpartum PMTCT clients who a) were aware that the method is a safe and effective contraceptive option for HIV-positive women and b) knew where they could receive the method. An intervention was introduced within maternal/ child health services in five public sector health centers in low-income peri-urban sections of Cape Town, South Africa. Intervention effectiveness was assessed through pre- and post-intervention cross-sectional interviews conducted with two samples of postpartum women exiting the clinic after receiving health services. To examine intervention implementation, in-depth interviews were conducted with providers in intervention facilities. Additional process data were collected through detailed implementation documentation.

### Study intervention

Working in consultation with the district health management team, the investigators designed a multi-faceted intervention that was informed by formative research previously completed by the investigators in South African PMTCT facilities. The earlier research revealed knowledge gaps on the part of health service providers and clients about use of long-acting and permanent contraceptive methods by women living with HIV [22]. The tested intervention focused on reinforcement of counseling on postpartum family planning delivered to PMTCT clients in antenatal, delivery, and child health services. Providers were trained to include the IUD and female sterilization among the methods promoted to clients, and clinical services for providing these methods were reinforced. Key intervention elements included the following (see Table 1, Column 1 for details):

• Provider training on reproductive health services for HIV-positive women

• Training on IUD insertion and removal

• Clinical supply management support

• Strengthened referral system for female sterilization

• On-the-job coaching and mentoring

• IEC materials and job aids

**Table 1:** Intervention elements as intended and related challenges during implementation

**Column 1: Intervention elements as intended**	**Column 2: Challenges encountered during implementation**
**Provider training on reproductive health for HIV-positive women**	
• Capitalized on provincial-wide training being conducted through a PEPFAR-sponsored initiative, independent of the study.	• Some trainees were not actually responsible for counseling clients; they were sent because they were the lowest ranking in the clinic, and their time was perceived as most expendable.
• 5-day curriculum covered the sexual and reproductive rights of women; prevention of unintended pregnancy as a means of reducing maternal to child transmission of HIV; contraceptive methods that are safe and effective for HIV-positive women; and the promotion of dual protection.	• Participants lacked fundamental knowledge and skills for family planning service delivery. Instructors had to quickly supplement curriculum with basic family planning information before covering service adaptations for HIV-positive women.
• Participants include providers responsible for antenatal care, child health services, and family planning services.	• These training needs were not anticipated and accounted for in advance because district management lacked readily accessible records about providers’ completed in-service training.
**IUD training**	
• Four days of theoretical classroom training led by a physician trainer.	• Managers recognized that higher ranking nurses would be most appropriate for the clinical training on IUD insertion, but they were reluctant to release them for the course given staff shortages.
• Immediately followed by clinical training and certification.	• Intervention was adjusted, dividing the 1-week training into two 2-day sessions to be held a few weeks apart.
	• A municipal strike caused a 2-month delay between the first and second sessions, necessitating thorough review of the earlier material.
	• To minimize providers’ time away from work, rather than offer the practical training to the group in a centralized location, it was conducted in the trainees’ home facilities with the support of a traveling trainer.
	• Few providers completed the practical portion of the IUD insertion training. Trainees struggled to recruit clients desiring the method, and many clients who were interested in the IUD failed to keep their appointments for insertion.
**Clinical supply management support**	
• Support for facilities in taking supply inventory and submitting orders to the sub-district health office.	• Facilities were slow in placing orders for IUD commodities and insertion equipment.
• Public sector health systems responsible for ensuring intervention sites adequately equipped to provide IUD services.	• Sub-district was not able to supply all insertion equipment requested.
	• Study staff supported sub-district and facility managers to resolve these issues by contacting and follow-up with sub-district management, arranging for equipment to be transferred between facilities, and in some cases transporting equipment.
**Strengthened referral system for female sterilization services**	
• Reinforced referral mechanisms between participating health centers and facilities offering sterilization services.	• Some providers reported that the required paperwork was burdensome.
• Both public sector reference facilities and those supported by the Association of Voluntary Sterilization South Africa (AVSSA), a non-governmental organization, serve as reference sites.	• Providers perceived the documentation as a requirement imposed by AVSSA, but in fact the documentation was consistent with provincial governmental policy.
	• Breaches in communication occurred between the public sector facilities and AVSSA sterilization service managers.
	• Some providers admitted that they did not make referrals because they lacked confidence in their ability to provide complete and accurate information about sterilization.
**On-the-job coaching and mentoring**	
• A medical officer, hired by the study, makes periodic visits to the health care facilities to offer facility-based coaching to support providers in applying knowledge and skills acquired during HIV-FP training.	• The coach visited facilities once or twice per month over the intervention period to reinforce information and skills that trained providers had been taught in project-supported courses, and she facilitated spread of knowledge to other clinicians who had not attended trainings.
	• She witnessed problems with provider morale, e.g., many providers complained that they felt overworked or that they were performing duties for which they had not been trained, including counseling on the IUD.
	• An important role of the coach was thus to provide encouragement to build confidence in performing new tasks and to offer general moral support about coping under difficult work conditions.
**IEC materials and job aids**	
• Facilities provided with the flipbook “Counseling Tool: Reproductive Choices for Clients with HIV,” adapted from *Decision-Making Tool for Family Planning Clients and Providers* and the *Reproductive Choices and Family Planning for People Living with HIV: Counselling Tool.*	• Providers used the flipbook inconsistently in delivering family planning counseling.
• Materials intended to be used by antenatal care, maternity, and child health service providers who serve PMTCT clients in the antenatal, peri-natal, and postpartum period.	• More time in training should have been dedicated to explaining purpose of educational materials, offering skill-building instruction on their use, and establishing the performance expectation that providers should use the flipbook routinely when counseling on family planning.
• Client pamphlets provided to present information on the full range of contraceptive options available in South Africa, including the IUD and sterilization.	• Clinics expressed a desire for additional material on the IUD. The study team produced and distributed copies of the “Balanced Counseling Strategy Plus” pamphlets developed by Population Council.

While the study team provided technical support for intervention implementation and oversight, the aim was to introduce an intervention package with potential for broad implementation within public sector services. Accordingly, intervention elements were incorporated into routine service delivery and depended on the systems and providers already in place in the participating public sector health facilities.

### Interviews with postpartum women

Before intervention implementation and 6 months after it was fully in place, we interviewed a total of 1077 women exiting child health services (538 pre-intervention and 539 post-intervention), with approximately equal numbers of women at each time period who had and had not enrolled in PMTCT services during their most recent pregnancy. Eligible participants were adult women who reported having received antenatal care and were seeking child health services for their infants at the 5 participating facilities. Different samples of clients were interviewed at each time period, as opposed to following a cohort over the 9-month intervention period, given the additional eligibility criterion of participants being no more than 6 months post-partum. During the two data collection periods, each lasting about 2 months, study participants were recruited in the waiting areas of the facilities; all clients were screened for eligibility and administered informed consent until the sample size was attained. Research assistants administered a structured questionnaire in either English or Xhosa, according to the participant’s choice, to collect information on socio-demographic characteristics, contraceptive use, reproductive history, fertility intentions, exposure to family planning counseling, and knowledge about the IUD and sterilization. Interviews took approximately 40 minutes, and participants were not compensated for their time.

This analysis focused on the PMTCT clients. Using Stata 10.0, we computed summary statistics separately for the pre-intervention and post-intervention samples, and then analyzed the difference between samples. Differences between samples were analyzed using rank sum test for continuous variables and chi-squared and Fisher’s exact test for categorical variables. The study was powered to assess whether at least 30% of PMTCT clients interviewed post-intervention had correct knowledge about the IUD and female sterilization, respectively, as captured by the primary outcome variables. Power estimates were based on post-intervention estimates instead of a change over time since it was assumed that clients in the pre-intervention sample would have negligible knowledge about those methods that were largely unavailable prior to the study.

### In-depth interviews with providers

Approximately 8 months after intervention introduction, a research assistant conducted individual in-depth interviews with 16 providers who had participated in the study-sponsored trainings. Open-ended questions explored providers’ attitudes toward and experiences with promoting voluntary contraceptive services to HIV-positive clients, with particular focus on IUD and female sterilization services. Questions examined providers’ views on the safety and appropriateness of those methods for women living with HIV. Providers were further asked about any approaches they take in assessing clients’ fertility desires and tailoring family planning accordingly. Other questions explored factors facilitating and impeding providers’ ability to promote use of the IUD and female sterilization, considering health system resources, the supports introduced through the study intervention, and clients’ needs and priorities. Interviews, conducted in English, were digitally recorded and took approximately 60 minutes. Providers were not compensated for their time. One lead investigator and an analyst read the transcripts and developed a coding scheme shaped by the interview guide. An analyst coded and analyzed transcripts using QSR International’s NVivo 9. To complete thematic analysis, the analyst prepared memos to capture the divergence and convergence of ideas emerging from the text data. The lead investigator reviewed the memos and introduced modest refinements in consultation with the analyst.

### Intervention tracking

The research team documented intervention implementation using a tracking tool. This document consisted of an Excel worksheet, similar to Table 1, with columns to record the intervention components, first as described in the research protocol and then as actually implemented. Investigators used a third column to record observations on challenges and considerations for future replication. Investigators filled in the tracking tool on an ongoing basis by abstracting information from trip reports, training reports, and team email correspondence.

### Ethics

The research was approved by the Health Science Faculty Human Research Ethics Committee of the University of Cape Town, the Western Cape Department of Health, and the Protection of Human Subjects Committee of FHI 360.

## Results

### Socio-demographic characteristics, reproductive history, and use of contraception

The demographic profiles of the participants sampled pre-intervention (herein “Pre-Int”) and post-intervention (“Post-Int”) were similar (Table 2). Participants, ranging in age from 18 to 53, were most commonly unmarried, in a steady relationship, and unemployed, with some secondary education. Significantly fewer Post-Int respondents reported being married or in a stable relationship compared to Pre-Int.

**Table 2:** Demographic profile of pre- and post-intervention sample of PMTCT clients

***Characteristic***		***Pre-int N = 265***	***Post-int N = 266***	***p-value***
		***n (%)***	***n (%)***	
**Age**	Median (IQR)	27 (23–32)	27 (24–31)	0.915
**Highest level of education**	No formal schooling	0 (0.0)	2 (0.8)	0.631
	Grade 1–7	19 (7.2)	14 (5.3)	
	Grade 8–12 without matric	171 (64.8)	180 (67.7)	
	Grade 12 with matric	66 (25.0)	62 (23.3)	
	Tertiary qualification	8 (3.1)	8 (3.0)	
**Current relationship status**	Married	88 (33.3)	69 (26.0)	0.007
	Single, stable relationship	166 (62.9)	167 (63.0)	
	Single, casual relationship	2 (0.8)	5 (1.9)	
	Single, no relationship	8 (3.0)	24 (9.1)	
**Employment status**	Paid Job (Yes)	66 (24.9)	64 (24.1)	0.841
	Paid Job (No)	199 (75.1)	202 (75.9)	

Most women at both time points reported their most recent pregnancy was unintended (Table 3). Of those interested in having another child, most wanted to wait at least three years or were unsure of desired timing. More than 80% of respondents in both samples reported current contraceptive use, primarily injectables. Fewer than 10% of women in both rounds had undergone sterilization, and just one woman, interviewed Post-Int, reported IUD use. Meanwhile, 77% of women Post-Int reported they wanted no future pregnancies. Asked if they would *consider* using the IUD, a third of the women Post-Int responded no; the most common reasons were that they lacked information about the method, were scared by it, or preferred sterilization. A quarter of the women Post-Int expressed no intention of undergoing female sterilization in the future, most commonly due to the permanency of the procedure or being afraid of it.

**Table 3:** Reproductive history, fertility desires, and contraceptive use among pre- and post-intervention samples

		***Pre-int N = 265***	***Post-int N = 266***	***p-values***
**Time since delivery (months)**	Median (IQR)	2.23 (1.30-3.47)	2.33 (1.40 -4.13)	0.080
**Number of living children**	Median (IQR)	2 (1–2)	2 (1–3)	0.164
		*n (%)*	*n (%)*	
**Last pregnancy intended**	Yes	101 (38.1)	110 (41.3)	0.488
	No	162 (61.1)	156 (58.7)	
**Would like another child**	Yes	30 (11.3)	39 (14.7)	0.018
	No	194 (73.2)	206 (77.4)	
	Unsure	41 (15.5)	21 (7.9)	
**Would consider using IUD in the future**	Yes	204 (77.0)	140 (52.6)	0.001
	No	44 (16.6)	88 (33.1)	
	Unsure	17 (6.4)	37 (13.9)	
	Already using	0 (0.0)	1 (0.4)	
**Would consider undergoing female sterilization in the future**	Yes	172 (64.9)	150 (56.4)	0.215
	No	57 (21.5)	66 (24.8)	
	Unsure	23 (8.7)	31 (11.7)	
	Already using	13 (4.9)	19 (7.1)	
**Currently using a family planning method**	Yes	238 (89.8)	222 (83.5)	0.032
	No	27 (10.2)	44 (16.5)	
*Among current family planning users*		*N = 238*	*N= 222*	
**Method currently used**	IUD	0 (0.0)	1 (0.5)	0.483
	Male sterilization	0 (0.0)	0 (0.0)	--
	Female sterilization	17 (7.1)	19 (8.6)	0.572
	Condoms	15 (6.3)	27 (12.2)	0.016
	Pills	2 (0.8)	4 (1.8)	0.436
	2 monthly injectable	48 (20.2)	38 (17.1)	0.402
	3 monthly injectable	167 (70.2)	153 (68.9)	0.771
*Among those desiring future pregnancy*		*N = 30*	*N = 39*	
**Desired timing of next child**	<12 months	0 (0.0)	0 (0.0)	0.469
	12–24 months	1 (3.3)	0 (0.0)	
	25–36 months	3 (10.0)	3 (7.7)	
	>36 months	13 (43.3)	23 (59.0)	
	Unsure	13 (43.3)	13 (33.3)	

### Knowledge and attitudes about the IUD and female sterilization

Table 4 presents the proportion of women demonstrating correct knowledge about the IUD and female sterilization as captured by the composite variables and their components. (Pre-Int participants were not asked about where they could access services since the research team understood those methods were largely inaccessible before the study.) Only 6% of women in the Post-Int survey answered correctly the four knowledge questions about the IUD, and 23% answered correctly the four knowledge questions about sterilization. The percentage of women correctly answering individual questions about the IUD declined between Pre-Int and Post-Int, while those correctly answering questions about female sterilization increased. Post-Int women were significantly more likely to know where HIV-positive women can undergo sterilization and that the procedure is safe for women using antiretrovirals. About one quarter of respondents Post-Int knew where they could access IUD services, compared to nearly two-thirds of women Post-Int who knew where they could access female sterilization services.

**Table 4:** Demonstrated knowledge and reported counseling received about IUD and female sterilization among pre- and post-intervention samples

	***Pre-int N = 265***	***Post-int N = 266***	**p-value**
	***n (%)***	***n (%)***	
**IUD**			
Composite outcome variable: Knows IUD is safe and effective for HIV + women and knows where to access it	Not computed	16 (6.0)	--
Elements in composite variable:			
• Knows HIV + women can use IUD	146 (55.1)	119 (44.7)	0.017
• Knows women on antiretroviral therapy can use the IUD	62 (23.4)	62 (23.3)	0.981
• Spontaneously states that a good thing about the IUD is that it is very effective against pregnancy	146 (55.1)	96 (36.1)	<0.0001
• Knows where she can access IUD	Not asked	75 (28.2)	--
**Female sterilization**			
Composite outcome variable: Knows female sterilization is safe and effective for HIV + women and knows where to access it	Not computed	60 (22.6)	--
Elements in composite variable:			
• Knows HIV + women can undergo sterilization*	201 (75.8)	223 (83.8)	0.022
• Knows women on antiretroviral therapy can undergo sterilization*	152 (57.6)	193 (72.6)	<0.0001
• Spontaneously states that a good thing about female sterilization is that it is very effective against pregnancy	113 (42.6)	141 (53.0)	0.107
• Knows where she can access female sterilization	Not asked	170 (63.9)	--
**Family planning counseling**			
Received counseling about family planning methods	128 (48.3)	148 (55.6)	0.091
*Among those receiving counseling*	*N = 128*	*N = 148*	
Provider counseled on IUD*	10 (7.8)	35 (23.7)	<0.0001
Provider counseled on female sterilization	74 (57.8)	96 (64.9)	0.230

*Indicates statistically significant changes in a favorable direction.

Roughly half the women interviewed at both time points reported having been counseled by a provider on family planning methods. Among those, the women reporting that they had been counseled on the IUD rose significantly from 8% Pre-Int to 24% Post-Int; women reporting counseling on female sterilization rose from 58% to 65%.

### In-depth interviews

The 16 interviewed providers shared perspectives on serving the contraceptive needs of both PMTCT clients and postpartum women in general. Most providers clearly supported the right of all women to make informed and voluntary choices about planning or avoiding pregnancy. Some shared accounts of reassuring HIV-positive women that they could maximize the chance of a healthy pregnancy and baby with a well-timed pregnancy. By contrast, very few providers expressed disapproval of their HIV-positive clients becoming pregnant.

Several providers stated that prior to the intervention, they were not aware that methods other than pills, injectables, and condoms were available in health services. Nearly all stated that it would be useful to expand the range of contraceptive options for all women, not just HIV-positive women. Providers described difficulties women face in using short-term methods consistently, mentioning, for example, that women discontinue injectables due to missed clinic appointments for re-injections. Others recounted clients who had discontinued injectables due to concerns about irregular bleeding or amenorrhea. Providers reported that women discontinuing injectables for either reason then tended to abandon contraceptive use completely due to perceived lack of alternatives.

Providers shared perspectives on the importance of expanding the contraceptive options for HIV-positive clients in particular. Concerning hormonal contraceptive methods, about a third of providers mentioned the need to consider drug interactions and to modify re-injection schedules if the client is on specific anti-retroviral drugs or tuberculosis treatment. Most providers stated the importance of encouraging their HIV-positive clients to use condoms; only a minority reported that they promote simultaneous use of condoms and another contraceptive. Nearly all providers mentioned challenges women face in using condoms consistently, leading some to acknowledge that condoms alone provide inadequate contraceptive protection for most women. A few providers, however, indicated that condoms were the “best” contraceptive method for HIV-positive women.

Providers expressed favorable views toward promoting the IUD and female sterilization, with most reporting that both are appropriate for HIV-positive women. Some providers said alternatives to short-acting methods could ease the burden of HIV clients who already have frequent clinic visits. Half the providers specifically mentioned that an advantage of the IUD is the quick return to fertility upon its removal. Some explained that this characteristic is particularly helpful to women on antiretroviral therapy who are highly motivated to avoid pregnancy until the point when their health status has improved and they are ready to conceive. Providers similarly had favorable views toward sterilization but were clear in stating that it was appropriate only for women who were certain they had achieved their desired family size. One provider admitted that before attending study-sponsored training, she had not thought of educating HIV-positive clients about sterilization, reasoning it would not be necessary since they already use condoms.

Providers offered insights on the study intervention that helped to explain its failure. Several mentioned that the hands-on clinical component of the IUD insertion training should have been longer to ensure providers mastered the skills and gained the confidence to deliver the service. A few respondents referenced as a training model their experience learning to perform Pap smears, whereby clinicians stayed for an extended period in a high-volume facility to obtain extensive, supervised experience in performing the new procedure. Some providers suggested whole-facility trainings on the IUD and sterilization to ensure that all providers promote these methods. About half the interviewed providers mentioned that the intervention would have been strengthened with expanded client education about family planning. Several described a need for community education about the IUD in particular to counter concerns about the method and to publicize its advantages. A number of providers specifically mentioned clients’ fears about the IUD, including the pain of insertion, the risk of the device shifting position or being expelled, or the partner feeling the string. One provider recounted her successful experience stimulating interest in the IUD when she had an opportunity to discuss the method on a talk radio program.

### Intervention tracking

Systematic documentation of intervention implementation captured information about the extent to which components were carried out as planned. Key intervention tracking findings are presented in Table 1, Column 2. The study team faced a host of challenges in implementing the intervention, some of which could be resolved with improved intervention design. For example, clearer criteria could be established to ensure that appropriate personnel are sent to trainings. Offering a condensed IUD service training to minimize providers’ absence from their work sites was the only option offered by district management, but it proved to be ineffective; instruction should have lasted longer and been conducted in a setting that provided trainees sufficient practical experience in insertion and removal techniques.

Other intervention shortcomings were rooted in health system constraints. Baseline provider capacity in basic family planning service delivery was weaker than anticipated; this fundamental deficiency needed to be addressed before training could be offered on IUD insertion and removal and the contraceptive considerations particular to HIV-positive women. Inadequacies of the health information system and limited time and resources for on-site supervision minimized district managers’ knowledge of the status of health service delivery within facilities and their influence over routine clinic operations. When the study-sponsored coach and research staff visited facilities, they sometimes noted high client loads for the staff available; at other times providers appeared to have a manageable number of clients to serve, but they demonstrated lack of motivation to take on additional responsibilities.

## Discussion

The study results indicate that the tested intervention failed to resolve PMTCT clients’ knowledge gaps constraining IUD and female sterilization use that were documented at baseline [23]. Use of female sterilization and the IUD by this sample of HIV-positive postpartum women was low at baseline and remained low following the intervention. Despite this study’s failure to show an intervention effect, the results are nonetheless informative. The high proportion of PMTCT clients at both time points reporting unintended pregnancies suggests that contraceptive services for HIV-positive women must be reinforced. The results show high reliance on short-acting contraception among women largely professing no desire for future pregnancies, indicating clear need to expand contraceptive options.

The process evaluation helps to explain the intervention’s ineffectiveness. In retrospect, some approaches for making it more robust are obvious. For example, community-level campaigns to overcome knowledge gaps and misconceptions [24] may have been a useful complement to clinic-based counseling. Such outreach was contemplated during intervention design but was dismissed since it was outside the range of practices sponsored by district health management. With renewed commitment by the Government of South Africa to community health worker programs, future initiatives may be able to use this resource for community mobilization. A longer intervention period may have allowed greater capacity-building among providers and full incorporation of enhanced practices into routine practice, which in turn may have produced more favorable results.

Other problems with intervention implementation rooted in health system weaknesses are not so readily resolved. Providers’ expressed support for promoting an expanded range of contraceptive methods was notably inconsistent with their observed delivery of such services. Training and follow-up coaching were apparently insufficient to overcome pervasive morale problems limiting providers’ motivation and confidence to take on additional responsibilities. The intervention was further constrained by weak management information systems. District managers did not have access to information needed to ensure, for example, that the right providers were sent to training or the required supplies were in place. The constraints documented in this study are consistent with those revealed by a recent qualitative investigation of service integration in South Africa [21]. For policies and guidelines to manifest in consistent provision of high quality integrated services, attention must be focused on health system-strengthening interventions to resolve service-level problems, including high client-staff ratios and insufficient provider knowledge and skills in sexual and reproductive health. Similar constraints will likely be encountered in other resource-poor settings when attempts are made to expand contraceptive options offered to PMTCT clients under typical circumstances [25].

The study’s chief limitation was the failure to implement the intervention fully as intended. However, since the main aim was to test the intervention under typical service delivery conditions, this outcome was informative. The absence of intensive support for the intervention, such as that offered in the context of clinical trials or donor-supported programming, produced insight into the results that could be experienced when a similar intervention is implemented under non-research conditions. A related strength is the detailed process documentation detailing how the intervention was implemented and the constraints encountered, responding to a call for greater process documentation in research to support identification of intervention elements warranting replication [26,27]. The study methods further serve as an example of the application of techniques used in implementation science, the discipline that emphasizes examination of health interventions under typical circumstances with careful examination of “fidelity,” a measure indicating extent to which components are implemented as intended [28]. Finally, the study would have been enhanced by qualitative interviews with clients to explore how HIV-positive women’s demand for long-acting and permanent contraceptive methods influenced the documented low uptake of those method.

## Conclusions

These study findings coincide with increased dialogue in South Africa about the integration of family planning and HIV/AIDS services, including the recent release of a new reproductive health policy emphasizing such linkages [29]. Future initiatives to expand contraceptive options for postpartum PMTCT clients in South Africa and elsewhere must include strategies to ensure that providers are equipped with the competence, the confidence, the material resources, and the motivation to offer additional services. Implementation strategies should include not only training and supervision, but health system reinforcements in areas like human resource management, commodity and supply management, and related management information systems. Past failures in serving the contraceptive needs of PMTCT clients are likely not rooted uniquely in the challenges associated with integrating family planning into HIV services; rather, they are also due to family planning service limitations more broadly. Making long-acting and permanent methods of contraception available to PMTCT clients must therefore be built on a foundation of improved provision of the full range of contraceptive options to all women.

## Competing interests

All authors declare they have no competing interests.

## Authors’ contributions

TH, JH, SC, and JM participated in the design of the study and the conception of the intervention. SC oversaw data collection and TP led intervention tracking. TH wrote the first draft of the manuscript. MG and DC performed the statistical analysis of quantitative data. TH led qualitative analysis. All authors participated in the critical revision of the manuscript, read and approved its content, and agree to be accountable for the reporting. All authors read and approved the final manuscript.
